# Prevalence and correlates of depressive symptoms in HIV-positive patients: a cross-sectional study among newly diagnosed patients in Yaoundé, Cameroon

**DOI:** 10.1186/1471-244X-13-228

**Published:** 2013-09-22

**Authors:** Rodrigue Minya L’akoa, Jean Jacques N Noubiap, Yixin Fang, Félicien Enyime Ntone, Christopher Kuaban

**Affiliations:** 1 Faculty of Medicine and Biomedical Sciences, University of Yaoundé I, Yaoundé, Cameroon; 2 Nguelemendouka District Hospital, Nguelemendouka, Cameroon; 3 Internal Medicine Unit, Edéa Regional Hospital, Edéa, Cameroon; 4 Department of Population Health, Division of Biostatistics, New York School of Medicine, New York, NY, USA; 5 Department of Psychiatry, Yaoundé Jamot Hospital, Yaoundé, Cameroon; 6 Faculty of Health Sciences, University of Bamenda, Bamenda, Cameroon

**Keywords:** Depressive symptoms, HIV patients, Cameroon

## Abstract

**Background:**

Depression is one of the most common neuropsychiatric complications of HIV disease, and in turn it is associated with worse HIV-related outcomes. Data on depression among HIV-infected patients in Cameroon are scarce. In this study, we report the prevalence and correlates of depressive symptoms among newly diagnosed HIV-infected patients in Yaoundé, Cameroon.

**Methods:**

Interviews were conducted with 100 newly diagnosed HIV-infected patients at three referral hospitals of Yaoundé. Depression was assessed using the nine-item Patient Health Questionnaire (PHQ-9). A positive depression screen was defined as PHQ-9 score greater than 9.

**Results:**

The overall prevalence of depressive symptoms was 63% (95% CI: 53.2 to 71.8), the majority having symptoms corresponding to moderate depression. Multiple logistic regression analysis showed that probable depressed patients were more likely than those who were not depressed to have had experience of alcohol abuse (OR: 19.03, 95% CI 3.11-375.85; p = 0.0083), and a 100 CD4 cells/mm3 fewer was associated with a 2.9 times increase of the odds of probable depression (95% CI 1.88-4.84; p < 0.0001).

**Conclusions:**

Our findings indicate a high prevalence of depressive symptoms in newly diagnosed HIV-infected patients in our setting, and their association with alcohol abuse and severe immunosuppression. This study also highlights the necessity to integrate mental health interventions into routine HIV clinical care in Cameroon.

## Background

The HIV/AIDS pandemic has caused far-reaching effects in low-income countries. Sub-Saharan Africa has been particularly hard hit. In 2010, 68% of all people living with HIV resided in Sub-Saharan Africa, a region with only 12% of the global population [1]. Sub-Saharan Africa also accounted for 70% of new HIV infections and 67% of AIDS-related deaths in 2010 [1].

Cameroon is one of the 22 priority countries in the World Health Organization’s *Global Plan towards the Elimination of New HIV Infections among Children and Keeping Their Mothers Alive*[2]. Situated in the Central Africa, Cameroon has the largest HIV/AIDS epidemic in this sub-region with an estimated HIV prevalence of 5.3%, 610,000 people living with HIV, 48,000 adults newly infected and 37,000 AIDS-related deaths reported in 2009 [3].

Depression is the most common neuropsychiatric complication of HIV disease [4]. HIV/AIDS and depression are projected to be the world’s two leading causes of disability by 2030 [5]. Reports on the actual prevalence of depression in HIV-infected persons have varied widely, from 22% [6] to 71% [7]. With 350 million people affected worldwide [8], rates of depression are roughly two times greater in people living with HIV than the general population (approximately 10% versus 5%), as determined by a meta-analysis of published studies [9]. Depression is associated with increased health care utilization [10], decreased quality of life [11], and increased suicide rate among patients in primary care [12]. Among people living with HIV/AIDS, depression increases the likelihood of HIV transmission [13], is associated with poor adherence to antiretroviral therapy (ART) [14] leading to virologic failure [15], and may independently increase HIV progression [16]. It is therefore crucial to identify patients with depression for an appropriate management. Unfortunately in Cameroon, the management of HIV/AIDS emphasizes on somatic aspects of the disease and neglects psychiatric manifestations, and depression is therefore underdiagnosed. This is essentially due to the fact that mental health is not effectively integrated into HIV/AIDS clinical care.

A recent study among HIV-infected patients on ART in a semi-urban centre in Cameroon reported that one in five participants met lifetime criteria for major depression disorders [17]. There are no other prior studies of depression prevalence among HIV patients in Cameroon. Our study aimed at determining the prevalence and correlates of depressive symptoms among newly diagnosed HIV-infected patients in Yaoundé, Cameroon.

## Methods

### Ethical considerations

This study was performed in accordance with the guidelines of the Helsinki Declaration and was approved by the National Ethics Committee of Cameroon (ethics approval N°008/CNE/SE/2011). All participants provided written informed consent.

### Study design and setting

We conducted an observational cross-sectional study in Yaoundé, Cameroon. Yaoundé is the administrative headquarters of the Centre Region and the political capital of the Republic of Cameroon. It is one of the two largest cities of Cameroon, with a population estimated at 2,000,000 inhabitants and characterized by a very great ethnic diversity; with almost all the 254 ethnic groups found in Cameroon represented [18].

The study was conducted between February and March 2011 in three HIV/AIDS treatment centers in Yaoundé which are among the pioneers in the fight against HIV infection in Cameroon. These centers are located in three referral hospitals: the Yaoundé Central Hospital that has the greatest pool of HIV-infected patients in Yaoundé, the Yaoundé Jamot Hospital that has the major centre for mental health in Yaoundé, and the Yaoundé General Hospital.

### Study population and sampling

The target population comprised of newly diagnosed HIV-patients. To be eligible, patients had to be diagnosed HIV-positive within the past six months, aged 18 years or more, speaking English or French and consenting to participate in the study. We excluded patients presenting with behavioral disorders that may impede the ability to answer the study questionnaire. Participants were identified by convenient and consecutive sampling of eligible patients who came for consultation on the days that the investigator conducted recruitment at each site. Recruitment was pursued until the target convenient sample size of 100 was reached.

### Instruments and measures

#### Background characteristics

Eleven background variables are: age, gender, education, area of residency (urban or rural), marital status, employment status, personal history of depression, family history of depression, alcohol abuse, last CD4 cell count and ART.

#### Screening for alcohol abuse

Alcohol abuse was diagnosed using the CAGE (*Cutting down, Annoyance by criticism, Guilty feelings, Eye-openers*) questionnaire [19]. This instrument includes four questions: (i) Have you ever felt you should cut down your drinking? (ii) Have people annoyed you by criticizing your drinking? (iii) Have you felt bad or guilty about your drinking? (iv) Have you ever had a drink first thing in the morning to steady your nerves or get rid of a hangover (eye-opener)? Alcohol abuse is diagnosed if there are at least two positive responses. The CAGE has been shown to have a sensitivity of 84.4% and a specificity of 93.1% to detect alcohol-related problems at this threshold of 2 positive responses [20].

#### Screening for depressive symptoms

Depression was assessed using the nine-item Patient Health Questionnaire (PHQ-9), a validated depression screening tool which is based on the Diagnostic and Statistical Manual of Mental Disorders, 4th Edition (DSM-IV) criteria [21]. Depression severity is based on the PHQ-9 scores, which ranges from 0 to 27. A PHQ-9 score of greater than 9 has a sensitivity of 83% and a specificity of 92% for major depressive disorder diagnosis [22]. In this study, a positive depression screen was defined as PHQ-9 score greater than 9; scores of 10 to 14 defined a moderate depression; scores of 15 to 19 defined a moderately severe depression; scores greater than 20 defined a severe depression. Some studies have addressed the issue of using screening instruments such as PHQ-9 to diagnose psychiatric disorder [23]. Therefore, given inability to report prevalence of depression, we report the prevalence of depressive symptoms.

### Data analysis

Data were coded, entered and analyzed using the Statistical Package for Social Science (SPSS) version 20.0 for Windows (SPSS, Chicago, Illinois, USA). We described quantitative variables using means with standard deviations (SD), and categorical variables using their frequencies and percentages. The *t*-test and the Chi-square test were used to compare quantitative and categorical variables respectively. Odds ratios (OR) with 95% confidence interval (CI) were used to evaluate the factors associated with depressive symptoms. The unadjusted ORs were calculated by univariate logistic regression. Furthermore, multiple logistic regression analyses were conducted based on five candidate models, which were compared using Akaike Information Criterion (AIC). The optimal model among the candidates is the one with the smallest AIC score [24]. This manuscript was written following STROBE guidelines for the reporting of observational studies [25].

## Results

### Background characteristics of study population

Among the 100 newly diagnosed HIV-infected participants, 52% were female; their ages ranged from 18 to 62 years with a mean of 40.4 years (SD = 10.4). Their CD4 cell counts ranged from 9 to 474 cells/mm^3^ with a mean of 234 (SD = 129). Sixty percent were living with a spouse, 54% were unemployed, and 76% were living in an urban area. The CAGE alcoholism screen was positive in 17% of the participants. Thirty-nine percent of participants had severe immunosuppression (CD4 cell count < 200). Five percent had had a previous episode of depression. Other characteristics are depicted in Table 1.

**Table 1:** Background characteristics of 100 newly diagnosed HIV-positive patients in Yaoundé, Cameroon

**Characteristic**	**Male (n = 48)**	**Female (n = 52)**	**P-value**
	**n (%) or mean (SD)**		
Age (years)	42.4 (10.3)	38.7 (10.3)	0.07
Marital status			
Single	16 (33.3)	24 (46.2)	0.19
Couple	32 (66.7)	28 (53.8)	
Education level			
Primary	16 (33.3)	20 (38.5)	0.76
Secondary	17 (35.4)	19 (36.5)	
University	15 (31.3)	13 (25)	
Employment status			
Employed	32 (66.7)	14 (27)	< 0.001
Unemployed	16 (33.3)	38 (73)	
Area of residence			
Urban	33 (68.7)	43 (82.7)	0.10
Rural	15 (31.3)	9 (7.3)	
Personal past history of depression			
Yes	3 (6.3)	2 (3.8)	0.50
No	45 (93.7)	50 (96.2)	
Family past history of depression			
Yes	2 (4.2)	0 (0)	0.22
No	46 (95.8)	52 (100)	
Alcohol abuse			
Yes	8 (16.7)	9 (17.3)	0.93
No	40 (83.3)	43 (82.7)	
Receive antiretroviral treatment			
Yes	19 (39.6)	21 (40.4)	0.93
No	29 (50.4)	31 (59.6)	
CD4 cells count (cells/mm^3^)	254 (116)	216 (139)	0.14

### Prevalence and correlates of depression

As shown in Table 2, 63% (95% CI: 53.2 to 71.8) of our study population had depressive symptoms, most of them having symptoms corresponding to moderate depression (46% of the entire sample, and 73% of the depressed ones).

**Table 2:** Prevalence of depressive symptoms among 100 newly diagnosed HIV-positive patients in Yaoundé, Cameroon

**Depression severity**	Frequency or percentage*	**95% CI**
No depression	37	28.2 – 46.8
Moderate depression	46	36.6 – 55.7
Moderately severe depression	16	10.1 – 24.4
Severe depression	1	0.2 – 5.4

* Since N = 100, frequencies and percentages are identical.

No depression: PHQ-9 scores less than 9; Moderate depression: scores of 10 to 14; Moderately severe depression: scores of 15 to 19; Severe depression: scores greater than 20.

Table 3 presents unadjusted odds of depression with those background characteristics. Under Bonferroni correction for multiple comparisons (i.e., using significance level 0.05/11 = 0.0045), both alcohol abuse (p = 0.003) and CD4 counts (p < 0.0001) are significantly associated with probable depression. These results are confirmed by logistic regression analyses summarized in Table 4. We compared five candidates models (1: includes all variables; 2: includes all variables suspected in the literature; 3: model 2 plus ART; 4: includes alcohol, CD4 cells count, and gender; 5: includes only alcohol and CD4 cells count) using Akaike Information Criterion (the smaller the better). It appears that model 1 was over-fit because of the largest AIC score associated with it. Moreover, the unusual changes in OR estimates from model 1 to 2 and 3 suggest that we should consider models sparser than model 1. It seems model 5, including only alcohol and CD4, was the best model among five candidates. Inference based on this model showed that probable depressed patients were more likely than those who were not depressed to have had experience of alcohol abuse (OR: 19.03, 95% CI 3.11-375.85; p = 0.0083), and a 100 CD4 cells/mm3 fewer was associated with a 2.9 times increase of the odds of probable depression (95% CI 1.88-4.84; p < 0.0001).

**Table 3:** Unadjusted correlates of depressive symptoms among 100 newly diagnosed HIV-positive patients in Yaoundé, Cameroon

**Measure**	**Total**	**Depression n (%)**	**OR (95% CI)**	**P-value**
Age (years)	100	-	0.99 (0.96 – 1.01)	0.34
Gender				
Male	48	26 (54.2)	Referent	
Female	52	37 (71.1)	0.48 (0.19 – 1.18)	0.078
Marital status				
Couple	60	38 (63.3)	Referent	
Single	40	25 (62.5)	0.96 (0.39 – 2.40)	0.93
Educational level				
University	28	18 (64.3)	Referent	
Less than university	72	45 (62.5)	0.93 (0.34 – 2.51)	0.86
Employment status				
Employed	46	29 (63)	Referent	
Unemployed	54	34 (63)	1.00 (0.41 – 2.44)	0.99
Area of residence				
Urban	76	46 (60.5)	Referent	
Rural	24	17 (70.3)	1.58 (0.53 – 4.82)	0.36
Personal past history of depression				
No	95	61 (64.2)	Referent	
Yes	5	2 (40)	0.37 (0.04 – 2.93)	0.26
Family past history of depression				
No	98	62 (63.3)	Referent	
Yes	2	1 (50)	0.58 (0.03 – 9.57)	0.70
Alcohol abuse				
No	83	47 (56.6)	Referent	
Yes	17	16 (94.1)	12.26 (1.57 – 259.21)	0.003
Antiretroviral treatment				
No	60	35 (58.3)	0.60 (0.23 – 1.52)	
Yes	40	28 (70)	Referent	0.23
CD4 cells count*	100	-	2.7 (1.79 – 4.72)	< 0.0001

* expressed by hundreds of cells/mm^3^. *OR* unadjusted odds ratio.

**Table 4:** Adjusted correlates of depressive symptoms among 100 newly diagnosed HIV-positive patients in Yaoundé, Cameroon

**Variables (referent)**	**Models**				
	**OR (p-value)**				
	**(1)**	**(2)**	**(3)**	**(4)**	**(5)**
Age	1.03 (0.28)	-	-	-	-
CD4 cell count*	3.30 (< 0.0001)	2.85 (< 0.0001)	3.12 (< 0.0001)	2.85 (< 0.0001)	2.9 (< 0.0001)
Gender (male)	2.42 (0.23)	1.93 (0.27)	2.05 (0.24)	1.95 (0.20)	-
Marital status (couple)	1.17 (0.78)	0.89 (0.83)	0.88 (0.82)	-	-
Educational level (university)	2.40 (0.24)	-	-	-	-
Employment status (employed)	0.49 (0.39)	1.06 (0.92)	0.95 (0.94)	-	-
Area of residence (urban)	2.25 (0.25)	-	-	-	-
Personal past history of depression (no)	0.55 (0.59)	-	-	-	-
Family past history of depression (no)	0.27 (0.61)	-	-	-	-
Alcohol abuse (no)	44.66 (0.003)	19.79 (0.01)	22.96 (0.0068)	20.0 (0.0084)	19.03 (0.0083)
Antiretroviral treatment (yes)	1.95 (0.28)	-	1.82 (0.31)	-	-
**AIC score**	109.40	103.24	104.17	99.3	98.9

* expressed by hundreds of cells/mm^3^. *OR* adjusted odds ratio, *AIC* Akaike Information Criterion.

## Discussion

Depression is a major problem in HIV-infected patients, because it can lead to poor adherence to ART, treatment failure, HIV progression and death [13-16]. Several years ago, it had been prominently reported that mental health must be integrated in global initiatives for HIV/AIDS, and that research on mental health and HIV should be a high priority, especially in less wealthy countries like Cameroon [26]. Unfortunately, there is still a scarcity of data on mental health in Cameroon. This study is only the second one on depression in HIV-infected patients in Cameroon. Accordingly, we sought to determine the prevalence and correlates of depressive symptoms among newly diagnosed HIV-infected patients in Yaoundé, Cameroon.

We found that 63% of our participants were probably depressed. In similar studies, Ouedraogo et al., Kaharuza et al. and Bhatia et al. respectively found depression prevalence of 51.3% in Burkina Faso, 47% in Uganda and 45% in USA [27-29]. Collaborating with these reports, our findings point out that depression is highly frequent among newly diagnosed HIV-infected patients. This high prevalence of depressive symptoms in our study population may be due to several factors. First, it may be reflective of psychological distress, precisely an adjustment reaction, due to recent notification of seropositivity. Supporting this hypothesis, Lyketsos et al. found that depression and adjustment disorders equally account for psychiatric morbidity among newly diagnosed HIV-patients in a medical outpatient HIV clinic [30]. On the contrary, other studies have shown only a modest increase in psychological distress immediately after notification of seropositivity, suggesting that adjustment does not play a major role in psychiatric morbidity at diagnosis of HIV infection [31]. Much more, Savetsky et al. reported that 71% of an urban cohort were depressed a mean of 840 days after diagnosis, which is beyond the timeframe of adjustment [7].

The high prevalence of depressive symptoms in our study may also be explained by severe immunosuppression in the participants. Thirty-nine percent of participants had severe immunosuppression (CD4 cell count < 200), and univariate and multivariate logistic regression showed a significant association between severe immunodepression and depressive symptoms. Indeed, it has been shown that severe immunodepression and HIV-illness are predictors of depression [32-34]. It is therefore crucial to adequately treat depression in newly diagnosed HIV-patients to impact ART adherence which will in turn enhance physical health and quality of life and subsequently prevent long-term depression.

Seventeen percent of our participants were screened positive for alcohol abuse during the past six months. Consistent with previous reports [35], alcohol abuse strongly correlates with depressive symptoms in our study population. Factors such as unemployment and single status have been shown to strongly contribute to depression in HIV-patients [28,29,36]. In our study these factors were not significantly associated with depressive symptoms, emphasizing the great psychological burden of the HIV status itself in our participants.

Regardless of its correlates, the magnitude of probable depression reported in our study emphasizes the need to incorporate the management of depression in HIV-care guidelines in Cameroon. In their study in Bamenda, Cameroon, Gaynes et al. have reported that identification and successful management of major depression by a health care professional was infrequent [17]. Studies in developing countries have shown that proper interventions, including a cognitive-behavioral group plan and community-driven group interpersonal psychotherapy, can reduce depressive symptoms and may lead to a better quality of life for patients with HIV or patients in regions with a high prevalence of HIV [37,38].

This study has some limitations. First, the study was conducted in an urban context, and patients from rural areas were in fact from the accessible rural outskirts of the town. Patients from isolated rural areas were not included in this study, and the impact of the place of residence (rural or urban) on the occurrence of depression was not well estimated. Secondly, the convenient sample size of 100 may also imply imprecision in our findings. However, our findings are consistent with several prior reports in similar settings. In addition, our data are from three referral centers for HIV/AIDS management and mental health in Yaoundé, thus supporting their generalizability especially in the urban context of Cameroon. Thirdly, we used high sensitivity and specificity diagnostic tools and criteria for depressive symptoms, but the PHQ-9 and the CAGE were validated in western settings and not in the Cameroonian HIV clinic settings. Questions related to the impact of depressive symptoms on function were not assessed. Finally, it would be very important to evaluate the impact of depression on the adherence to ART in Cameroon, since this data is completely lacking. Indeed, all the studies on factors associated with adherence to ART in Cameroon, surprisingly, did not assess depression [39].

## Conclusions

Overall our findings indicate a high prevalence of depressive symptoms in newly diagnosed HIV-infected patients in Yaoundé, Cameroon, and their association with alcohol abuse and severe immunodepression. This highlights the need of efficient mental health interventions to be integrated into routine HIV clinical care in Cameroon. In this way, early screening and treatment of depression must be a major objective, since it may improve linkage to and retention in HIV care centers.

## Competing interests

The authors declare that they have no competing interests.

## Authors’ contributions

RML designed the study, collected data, contributed to data analysis and revised the manuscript. JJNN designed the study, analyzed and interpreted data, drafted and revised the manuscript. YF contributed to data analysis and revised the manuscript. FEN contributed to the study design, data collection and revised the manuscript. CK supervised the study at each step. All the authors approved the final version of the manuscript.

## Pre-publication history

The pre-publication history for this paper can be accessed here:

http://www.biomedcentral.com/1471-244X/13/228/prepub
